# Complete genome sequence of *Plesiomonas shigelloides* strain NBPZ-032, isolated from the intestine of Chinese rice-field eels (*Monopterus albus*)

**DOI:** 10.1128/mra.00914-25

**Published:** 2025-12-10

**Authors:** Wei Chen, Xinbo He, Yueying Wang, Ling Chen, Min Yong, Xiaoyan Liu, Lei Zhu

**Affiliations:** 1 National Biopesticide Engineering Technology Research Centre, Hubei Biopesticide Engineering Research Centre, Hubei Academy of Agricultural Sciences 117996, Wuhan, People's Republic of China; University of Maryland School of Medicine, Baltimore, Maryland, USA

**Keywords:** *Plesiomonas shigelloides*, Chinese rice-field eels, *Monopterus albus*, complete genome

## Abstract

We presented the complete genome of *Plesiomonas shigelloides* strain NBPZ-032 isolated from the intestine of Chinese rice-field eels (*Monopterus albus*), of which the total length is 3,711,769 bp and contains 3,127 protein-coding sequences.

## ANNOUNCEMENT

Chinese rice-field eels (*Monopterus albus*) hold substantial economic significance and are integral to regional food cultures in China, with annual output exceeding 300,000 tons—nearly half from Hubei Province (1). *Plesiomonas shigelloides*, a gram-negative, non-spore-forming, rod-shaped, facultative anaerobic bacterium, and aquatic animal pathogen (2), has poorly understood pathogenicity in this species (3).

Strain NBPZ-032 was isolated in April 2025 from the intestine of an artificially cultured (controlled aquaculture) Chinese rice-field eel in Xiantao, Hubei, China (113.4430°N, 30.3278°E). The whole intestinal tract was aseptically excised, weighed 10g and homogenized in 10 mL sterile phosphate-buffered saline (PBS), serially diluted using PBS and spread on lysogeny broth (LB) agar. After incubation (HP150S, Ruihua, China) at 30°C for 2 days, single colonies were purified via three rounds of streaking on LB plates, then cultured in LB at 30°C to the middle logarithmic phase by spectrophotometer (TU-1810, PERSEE, China). Genomic DNA was extracted using the Blood & Cell Culture DNA Mini Kit (Cat#13323, QIAGEN, Germany) according to the manufacturer’s instructions with purity/quantification via NanoDrop One UV-Vis spectrophotometer (Thermo Fisher Scientific, USA) and Qubit 3.0 Fluorometer (Invitrogen, USA). The long- and short-read sequencings were performed from the same DNA preparation. Long-read sequencing used size-selected long DNA fragments (>10 kb) by the PippinHT system (Sage Science, USA). The DNA library was prepared using a Ligation Sequencing Kit V14 (Cat#SQK-LSK-114; Oxford Nanopore Technologies, Ltd. [ONT], Oxford, UK) without DNA shearing and was sequenced with a Nanopore PromethION sequencer (ONT) on a R10.4.1 flow cell (FLO-MIN114). Base-calling and barcode segmentation, adapter sequences removal for the raw sequences were performed using Guppy v.5.0.16 (4). Raw data (97,502 reads, 1,144,952,152 bp in total) were generated with a reads-mean-length of 11,742.86 bp and N50 was 24,867 bp, and 97,497 reads (1,144,951,241 bp) were yielded after base-calling and adapter trimming. For short-reads sequencing, genomic DNA was randomly fragmented by Covaris LE220 focused ultrasonicator (USA), size-selected to 200400 bp using MGIEasy DNA Clean Beads (Cat#1000005279, MGI Tech Co., Ltd., China). DNA library was prepared following the MGIEasy FS PCR Free DNA library preparation set (Cat#1000013455, MGI). Sequencing was performed on a DNBSEQ-T7RS platform (MGI), using 150 nucleotide length paired-end reads. Raw data (7,247,348 reads, 1,087,102,200  bp in total) were processed using the FASTQ preprocessing program fastp v.0.23.1 (5) to trim adapters and low-quality data, yielding 6,975,838 clean reads (1,045,312,346  bp in total).

The complete genome (3,711,769 bp length in total) was assembled into two circular replicons through Unicycler v.0.5.0 (6): a 3,359,540 bp circular chromosome (52.01% GC content, 296.61× depth) and a 352,229 bp circular plasmid (49.75% GC content, 280.43× depth). Phylogenetic analysis using the Type (Strain) Genome Server revealed a close evolutionary relationship with *P. shigelloides* type strain NCTC 10360 (NCBI assembly accession no. GCA_900087055), supported by a digital DNA–DNA hybridization value of 75.6% calculated using GGDC formula 2 (7). Annotation via NCBI PGAP v.6.10 (8) identified 3,127 protein-coding sequences, 120 tRNA genes, and 40 rRNA genes. In this study, default parameters were used except where otherwise noted.

## Data Availability

This BioProject has been deposited at GenBank under the accession number PRJNA1277015. The whole genome is available in GenBank under the accession numbers CP195150 and CP195151. The raw sequencing data were deposited in the Sequence Read Archive (SRA) database under the accession numbers SRR33999113 (MGI) and SRR33999114 (ONT).
